# Trade-Off between Growth and Carbohydrate Accumulation in Nutrient-Limited *Arthrospira* sp. PCC 8005 Studied by Integrating Transcriptomic and Proteomic Approaches

**DOI:** 10.1371/journal.pone.0132461

**Published:** 2015-07-21

**Authors:** Orily Depraetere, Frédéric Deschoenmaeker, Hanène Badri, Pieter Monsieurs, Imogen Foubert, Natalie Leys, Ruddy Wattiez, Koenraad Muylaert

**Affiliations:** 1 KU Leuven campus Kortrijk, Laboratory Aquatic Biology, E. Sabbelaan 53, 8500, Kortrijk, Belgium; 2 Department of Proteomic and Microbiology, Research Institute for Biosciences, University of Mons, Place du Parc 20, 7000, Mons, Belgium; 3 Expert Group for Molecular and Cellular Biology MCB, Belgian Nuclear Research Center SCK.CEN, 2400, Mol, Belgium; 4 KU Leuven campus Kortrijk, Research Unit Food & Lipids, Department of Molecular and Microbial Systems Kulak, Etienne Sabbelaan 53, 8500, Kortrijk, Belgium; Mount Allison University, CANADA

## Abstract

Cyanobacteria have a strong potential for biofuel production due to their ability to accumulate large amounts of carbohydrates. Nitrogen (N) stress can be used to increase the content of carbohydrates in the biomass, but it is expected to reduce biomass productivity. To study this trade-off between carbohydrate accumulation and biomass productivity, we characterized the biomass productivity, biomass composition as well as the transcriptome and proteome of the cyanobacterium *Arthrospira* sp. PCC 8005 cultured under N-limiting and N-replete conditions. N limitation resulted in a large increase in the carbohydrate content of the biomass (from 14 to 74%) and a decrease in the protein content (from 37 to 10%). Analyses of fatty acids indicated that no lipids were accumulated under N-limited conditions. Nevertheless, it did not affect the biomass productivity of the culture up to five days after N was depleted from the culture medium. Transcriptomic and proteomic analysis indicated that *de novo* protein synthesis was down-regulated in the N-limited culture. Proteins were degraded and partly converted into carbohydrates through gluconeogenesis. Cellular N derived from protein degradation was recycled through the TCA and GS-GOGAT cycles. In addition, photosynthetic energy production and carbon fixation were both down-regulated, while glycogen synthesis was up-regulated. Our results suggested that N limitation resulted in a redirection of photosynthetic energy from protein synthesis to glycogen synthesis. The fact that glycogen synthesis has a lower energy demand than protein synthesis might explain why *Arthrospira* is able to achieve a similar biomass productivity under N-limited as under N-replete conditions despite the fact that photosynthetic energy production was impaired by N limitation.

## Introduction

Microalgae are considered to be a promising feedstock for the production of biofuels [1,2]. Most research on microalgal biofuels is focusing on production of lipids for their conversion into biodiesel. However, many species of microalgae are known to accumulate carbohydrates to a much greater extent than lipids. Carbohydrates can be converted into bio-ethanol using alcoholic fermentation or into bio-methane using anaerobic digestion [3]. This carbohydrate route for biofuel production is more efficient in terms of light energy conversion into bio-energy feedstocks than the classical biodiesel route [4]. Approximately 6.3 moles ATP per C are required for the production of lipids in microalgae as opposed to only 4.2 ATP per C for carbohydrates (*i*.*e*., a difference of 53%), which corresponds to a 50% higher energy demand. On the other hand, lipids only yield 41% more energy than carbohydrates in thermal oxidation processes (*i*.*e*., as biodiesel), and only 32% more in biochemical oxidation (*i*.*e*., in bio-methane or bio-ethanol formation) [4].

The cyanobacterium *Arthrospira* is the photosynthetic microorganism that is cultured at the largest scale. Although cyanobacteria are unrelated to microalgae, their production and applications are comparable. The global production of *Arthrospira* is estimated at 10 thousand tons dry biomass per year, and represents 50% of the total microalgal biomass production [5]. Cultivation of *Arthrospira* is relatively easy compared to other microalgae because contamination of cultures is easily avoided and the biomass can be harvested using simple microstrainers [1]. Since the accumulation of lipids in *Arthrospira* is low, as in other cyanobacteria, it is not attractive for biodiesel production [6]. Nevertheless, this microorganisms is able to accumulate up to 70% of carbohydrates in its biomass under nitrogen (N) limitation, mostly as glycogen granules [7–12]. The advantages of *Arthrospira* cultures (*i*.*e*., ease of cultivation and strong carbohydrate production) make *Arthrospira* a highly attractive candidate for production of carbohydrate-based biofuels in the near term.

When *Arthrospira* is cultured in regular medium containing non-limiting N concentrations, the cells produce large quantities of proteins (up to 63% of the biomass) [13]. When N is depleted from the culture medium, the concentration of proteins in the biomass is reduced, while the concentration of carbohydrates increases [14]. Proteins are crucial for the cells, because they form the main resource acquisition mechanisms of the cell being essential for photosynthetic C-fixation in the light, and thus the production of new biomass. Therefore, N limitation not only causes carbohydrate accumulation, but also slows down the rate of biomass production [15,16]. As a result of this trade-off between growth and carbohydrate accumulation, the total carbohydrate yield of a N-limited culture may decrease despite the fact that the carbohydrate content of the biomass increases [17]. In[10], for instance, they observed the highest carbohydrate concentration in the biomass in a culture lacking nitrate in the medium, but the carbohydrate yield of the culture was higher at intermediate nitrate concentrations (3 mM).

In order to maximize the production of carbohydrates and to minimize the trade-off with biomass production by an *Arthrospira* culture, it is important to understand the metabolic changes that occur under N-limited growth (*i*.*e*., investment in storage compounds like carbohydrates and degradation of resource acquisition mechanisms). The combination of genome and proteome tools offer a huge potential to provide insight into the metabolic changes that occur during N-limited growth [18–20]. Metabolic changes induced by N limitation have already been characterized in several species of cyanobacteria (*e*.*g*., *Synechococcus* and *Prochlorococcus* [21,22], *Microcystis* [23], *Anabaena* [24] or *Synechocystis* [25–27]). These studies documented major changes in the metabolism following the induction of N limitation, including up-regulation of N-acquisition genes and down-regulation of photosynthesis.

The annotated genome sequence of two *Arthrospira* strains were published for the first time in 2010 by Janssen *et al*. (*Arthrospira* PCC 8005) [28] and by Fujisawa *et al*. (*Arthrospira platensis* NIES-39) [29]. Other strains were sequenced in 2012 (*Arthrospira platensis* C1 PCC9438 [30]) and 2014 (*Arthrospira platensis* strain Paraca [31]). These genome investigations paved the way for studies of the response of *Arthrospira* to various stresses using transcriptomic and/or proteomic studies (*e*.*g*., response to light/dark cycle [32], temperature stress [33] or salt stress [34]). So far, a few studies have investigated the response of *Arthrospira* to N limitation on the proteomic level [14]. In that study, *Arthrospira* cells were transferred from N-replete conditions to a complete N-free medium. This situation is different from a N-limited culture, where the cells experience gradually declining N concentrations in their medium.

The goal of this study was thus to investigate the response of *Arthrospira* strain PCC 8005 to N limitation integrating transcriptomic and proteomic analyses. Changes in the metabolism as evaluated from transcriptomic and proteomic analysis were compared with changes in protein and carbohydrate content and in the photosynthetic mechanisms. We specifically wanted to evaluate the trade-off between carbohydrate accumulation and biomass productivity. To our knowledge, only two studies have compared the effect of an environmental stress factor on both transcriptomic and proteomic level in cyanobacteria, and both studies were on *Synechocystis* sp. PCC 6803 [26,35].

## Materials and Methods

### 1. *Arthrospira* cultivation

The *Arthrospira* strain PCC 8005 (Pasteur collection, France) was maintained axenic in sterile 250 mL Erlenmeyer flasks on a rotary shaker (120 rpm). The cultures were incubated at 30°C under a continuous irradiance of ± 39 μmol photons m^-2^ s^-1^. The culture medium used was Zarrouck’s medium as modified by Cogne [36].

To evaluate the effect of N limitation on productivity, biomass composition and the transcriptome and proteome of *Arthrospira* strain PCC 8005, two treatments with three replicates were compared: a control treatment with non-limiting N concentration (100 mg N L^-1^) and a N-limited treatment (20 mg N L^-1^). Preliminary experiments had shown that N was depleted after 5 days in the N-limited treatments while it remains well above limiting levels up to 10 days in the control treatment (> 35 mg N L^-1^).

A first experiment was set up to evaluate the influence of N limitation on biomass composition and productivity. Every day except for days 6 and 7, the biomass concentration was estimated from optical density measurement at 750 nm and the culture medium was sampled to analyze dissolved nutrients. Samples for dissolved nutrients were filtered over a 0.45 μm cellulose nitrate filter and stored frozen. At the end of the experiment on day 10, dry weight was determined gravimetrically after filtration of a known volume on a pre-weighed microfiber GF/C filter. The remaining biomass was harvested using 20 μm nylon mesh, rinsed once with de-ionized water, freeze-dried and kept frozen at -20°C for further analysis.

Dissolved phosphate and nitrate concentrations in the culture medium were measured using standard protocols (malachite green for phosphate [37]; 2.6-dimethylphenol method for nitrate [38]). The N and P content of the biomass was measured as phosphate and nitrate ions after biomass lysis using the acid persulphate digestion method [39]. Carotenoids and chlorophyll were measured spectrophotometrically according to [40] and phycocyanin according to [41]. The amounts of proteins and total carbohydrates were respectively evaluated by Bradford assay [42] and phenol-sulphuric acid method [43]. Lipid content and fatty acid profile were determined according to the modified direct trans-esterification method [44,45].

A second experiment was carried out to investigate the metabolic response to N limitation using transcriptomic and proteomic analyses. Four replicate N-limited and control *Arthrospira* strain PCC 8005 cultures were set up under exactly the same conditions as described above. Samples were collected daily during 10 days to estimate the biomass density by optical density measurement (750 nm) and to analyze dissolved nitrate concentrations after filtering over a 0.45 μm cellulose nitrate filter. On day 7, biomass samples for transcriptomic and proteomic analysis were collected by centrifugation (10 000 g, 15 minutes, 4°C) and stored frozen (-80°C).

### 2. Transcriptomic analysis

RNA extraction was performed as described in [46]. Prior to RNA extraction, the biomass sample was mixed with 1 mL Trizol to prevent RNA degradation during defrosting (Invitrogen). Cells were lyzed by a temperature shock procedure (5 minutes at 95°C followed by 5 minutes in ice bath). The released RNA was separated from the cell debris by centrifugation (10 000 g, 10 minutes, 4°C). RNA was purified using the Direct-zol RNA miniprep 2050 kit following the manufacturer's instructions (Zymo Research). DNA was removed using the Ambion TURBO DNA-free kit following the manufacturer's instructions (Life Technologies). The RNA was concentrated using Zymosearch RNA Clean & Concentrator-25 (Laborimpex, Brussels, Belgium). The quantity and purity of the RNA was assessed using a NanoDrop ND-1000 Spectrophotometer (Thermo Scientific). The quality and integrity of RNA was checked with the Bioanalyzer 2100 (Agilent Technologies) according to manufacturer's instructions. Absence of DNA was confirmed by PCR with universal 16S rRNA primers.

The microarray was designed by Roche NimbleGen based on version 3 of the full genome of *Arthrospira* PCC 8005 (692 contigs, ~6.8 Mbp), sequenced by Genoscope (Team of Dr. Valerie Barbe) [28]. A 12x135k tiling array *Arthrospira* HX12 was designed with probes ranging from 50 up to 72 nucleotides (mean length of 53 nucleotides) and on average over 34 nucleotides. The total of 135 367 probes were mapped back to the improved version 5 of the *Arthrospira* PCC 8005 (EMBL database GCA_000176895, CAFN1000000), which includes 5853 gene coding sequences and 3142 intergenic regions.

The RNA extracts (25 ng) were converted into a cDNA library using the Complete Whole Transcriptome Amplification WTA2 kit according to the instructions of the manufacturer (Sigma-Aldrich). The cDNA was labelled with Cy3 nonamer primers and Klenow polymerization and 2μg Cy3-labeled cDNA was hybridized to the *Arthrospira* HX12 array for 18 hours at 42°C. The arrays were washed and scanned in a MS 200 scanner (Roche-Nimblegen). Raw data files (Pair and XYS files) were obtained from images using DEVA software (Roche-Nimblegen) for further analysis [46].

Raw data files were pre-processed using the “Oligo” package (version 1.24) in BioConductor (version 2.12 / R version 3.0.1). Pre-processing included i) background correction based on the Robust Multichip Average (RMA) convolution model [47], ii) quantile normalization to make expression values from different arrays more comparable [48], and iii) summarization of multiple probe intensities for each probe set to one expression value per gene using the median polish approach [47]. To evaluate changes in gene expression between N-replete and N-limiting conditions, the Bayesian adjusted t-statistics were used as implemented in the “LIMMA” package (version 2.18.0) [49]. *p*-values were corrected for multiple testing using the Benjamini and Hochberg’s method to control for false discoveries [50]. Transcripts were considered as significantly differentially expressed when the corresponding adjusted *p*-value was less than 0.05 and their absolute fold change (FC) was equal or larger than 1 for up-regulated genes, and equal or less than -1 for the down-regulated ones [46].

### 3. Proteomic analysis

Proteomic analysis was carried out as reported in [14]. Briefly, proteins were extracted in 6M guanidinium chloride pH = 8.5 (Lysis buffer of ICPL kit (SERVA, Germany)) by ultrasonication (3x10 seconds, 20% amplitude, U50 IKAtechnik). Proteins were recovered by centrifugation (13.2x10^3^ rpm, 15 minutes, 4°C), and 5μg of proteins (Bradford, 1976) were submitted to a label-free differential proteomic analysis. Previously, proteins were reduced and alkylated according to the instructions of the manufacturer (Serva kit protocol). Proteins were precipitated with acetone overnight, and then dissolved in 40μl of 50mM NH_4_HCO_3_ (v/v, pH = 8.5) containing 1μg of trypsine (Promega V51 11). Samples were incubated at 37°C overnight, and trypsinization was stopped with formic acid (0,1% final v/v). Trypsic peptides (0.8μg/μL final concentration, v/v) were separated on reverse phase column (length: 25cm, diameter: 75μm, particles: 3μm, outlet: 300nL/min, PepMap C18, Dionex), and submitted to an ACN gradient (4 to 35%, v/v) for 120 minutes. Previously, the column was equilibrated with 4% ACN during 20 minutes, and each peptide elution was followed by a wash step (90% v/v ACN, 10 minutes). Online MS analysis was performed with *ABSCIEX* 5600 *TripleTOF*. Peptide mass spectra were acquired in DDA mode. One MS spectrum (m/z: 400–1500; acquisition time: 0.5 seconds) was acquired followed by 50 MS/MS (m/z: 100–1800, acquisition time: 0.05 seconds) spectra under HS mode for each acquisition cycle. The 50 precursors with at least 200 counts were selected, and were submitted to a CID with nitrogen gas. The selected precursors were excluded after 1 MS/MS spectrum for 30 seconds. To maintain average mass error below 10 ppm during analysis, calibration of TOF analyzer was automatically performed with trypsic peptides of β-galactosidase from *Escherichia coli* after every 4 samples.

ProteinPilot (v4.5) was used for protein identification, which was performed against a local copy of the *Arthrospira* PCC 8005 genome version 5 using the Paragon algorithm (4.0.0.0, 459). Search parameters were defined as trypsin for digestion enzyme, cysteine alkylation for iodoacetamide, and thorough ID search effort was processed. ID focus also considered the biological modifications.

Protein quantification was performed using Skyline software (v2.0) as previously reported [51,52]. Previously, identified protein list was filtered to obtain an FDR of 1% at protein level. Background proteome was built according to the protein database deduced from version-5 genome sequence of *Arthrospira* PCC 8005. Raw files were imported into Skyline to extract MS1 precursors of each peptide identified in the MS/MS spectral libraries. The three isotope peaks (M, M+1, M+2) of the isotopic envelope for each peptide precursor were extracted from the XICs, and used for the quantification of proteins. 2 minutes of time window defined the prediction parameters, and the modification of peptides included carbamidomethylation (C) as fixed modifications, oxidation of methionine and deamination of asparagine and glutamine as variable modifications.

Finally, a report was generated summarizing each area of each peptide calculated in natural logarithmic space. Normalization of data was performed according to the median of the replicates, and fold change was calculated as the ratios of differential expression between N-limited and control condition at day 5 of culture. Significance of fold changes between our experimental conditions (control and N-limited) was evaluated through student test with statistical threshold at 5% (*p-*value < 0.05). Only proteins which exhibited a significant fold change (*p-*value < 0.05) and were identified by at least 2 peptides were retained for biological interpretation. All peptides from each retained protein were manually validated. Protein classification was determined with COG automatic classification obtained from Genoscope and the specific activity of proteins considered was elucidated with KEGG database.

## Results and Discussion

### 1. Effect of N limitation on growth and biomass composition

**Fig 1:**
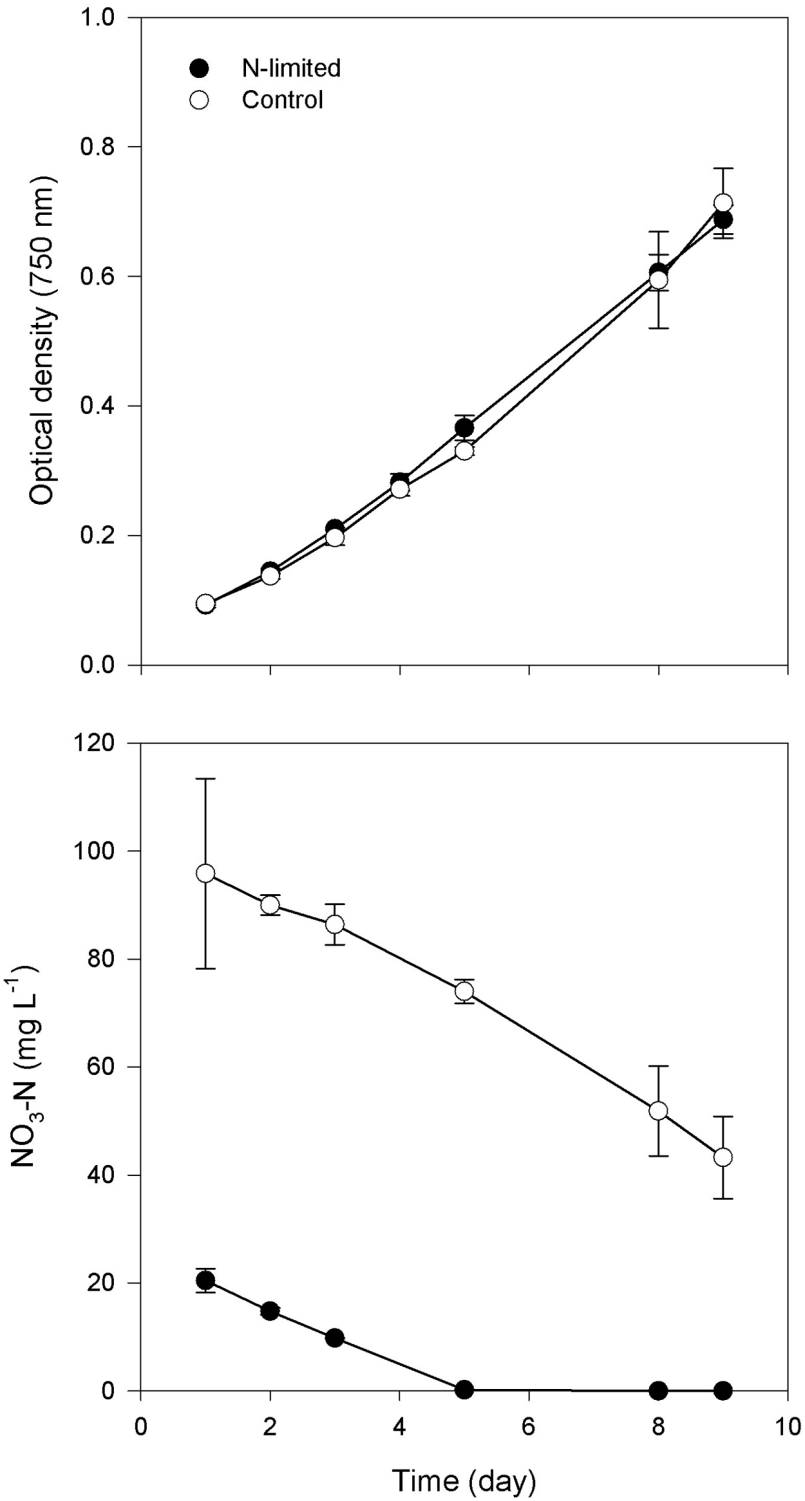
Changes of biomass density evaluated by measuring the optical density at 750 nm and NO_3_-N concentration (mg L^-1^) in the culture medium of the control (○) and N-limited *Arthrospira* sp. PCC 8005 (●).

To evaluate the impact of N limitation on *Arthrospira* at both organism (*i*.*e*., growth) and molecular level (*i*.*e*., biomass composition, transcriptome and proteome level), we compared cultures of *Arthrospira* in N-limited and N-replete medium. N was depleted on day 5 in the N-limited medium, while it remained above limiting threshold in the control medium (Fig 1). Based on OD_750_ measurement, growth did not significantly differ between the N-limited and the control culture up to day 10. The final biomass concentration (day 10) as estimated from dry weight measurements also did not differ significantly between the control and N-limited cultures (Table 1).

**Table 1:** Dry weight (g L^-1^) and composition (%) of control and N-stressed *Arthrospira* sp. biomass at the end of the experiment (day 10).

Parameter	Control	N stress
dry weight (g L^-1^)	0.59±0.06 ^a^	0.66±0.04 ^a^
total sugars (%)	13.47±0.83 ^a^	73.70±3.10^b^
proteins (%)	36.53±5.71 ^a^	10.44±1.66 ^b^
phycocyanin (%)	8.00±1.53 ^a^	1.34±0.20 ^b^
chlorophyll (%)	2.08±0.15 ^a^	0.66±0.04 ^b^
carotenoids (%)	0.39±0.01 ^a^	0.15±0.01 ^b^
nitrogen (%)	9.58±0.59 ^a^	3.29±0.16 ^b^
phosphorus (%)	0.78±0.05 ^a^	0.39±0.02 ^a^
FAME (%)	4.84	1.67

FAME, fatty acid methyl esters. Statistical analyses were performed using Sigma-plot 11 (Systat Software, Inc.). Before evaluating the results with one-way analysis of variance (ANOVA), normality of the data was determined with the Shapiro-Wilk normality test. To analyze the pairwise differences, a Tukey’s post-hoc test was used. The significance level of statistical analyses (*p-*value) was set at 5%.

^a,b^ Different letters indicate statistical difference.

In agreement with previous studies of N-limited *Arthrospira* cultures [14], the biomass from the N-limited culture had a much higher carbohydrate concentration and a much lower protein concentration than the control culture. Fatty acids were not accumulated in the N-limited culture. The N-limited cultures experienced chlorosis, as was evident from the lower concentration of photosynthetic pigments phycocyanin, chlorophyll *a* and carotenoids in the biomass.

### 2. Comparison between proteomic and transcriptomic data

Of the 5853 gene coding sequences, 4629 could be matched with a known function. Of these, 322 were significantly down-regulated, while 319 were significantly up-regulated. It should be noted that another 76 genes of unknown function were significantly up-regulated while 66 were down-regulated in the N-limited treatment. In the proteomics analysis, 4589 proteins were identified and 4142 of those could be matched with a known function. Among those, 21 proteins had a lower abundance and 127 proteins had a higher abundance in the N-limited treatment. Another 34 proteins of unknown function had a higher abundance in the N-limited treatment.

32 genes were up-regulated at both protein and transcript level, while 5 genes were down-regulated at both protein and transcript level. 12 genes exhibited an opposite response at the protein and transcript level. In general, genes encoding glycolysis/gluconeogenesis, TCA cycle enzymes and N metabolism were significantly up-regulated both at the protein and transcript level. On the contrary, genes related to photosynthesis (chlorophyll synthesis, photosystem I and II, ATP synthesis, phycobilisome linker polypeptide, phycocyanin synthesis and carbon fixation) as well as protein synthesis (ribosomal genes) were significantly down-regulated at the transcript level but did not display any response on the protein level.

For glycogen synthesis, we observed an up-regulation at the protein level but not at the transcript level. Discordant changes between proteomics and transcriptomics studies have also been observed in *Synechocystis* sp. PCC6803 under N-limitation [26].

### 3. Up-regulation of N acquisition mechanisms

**Fig 2:**
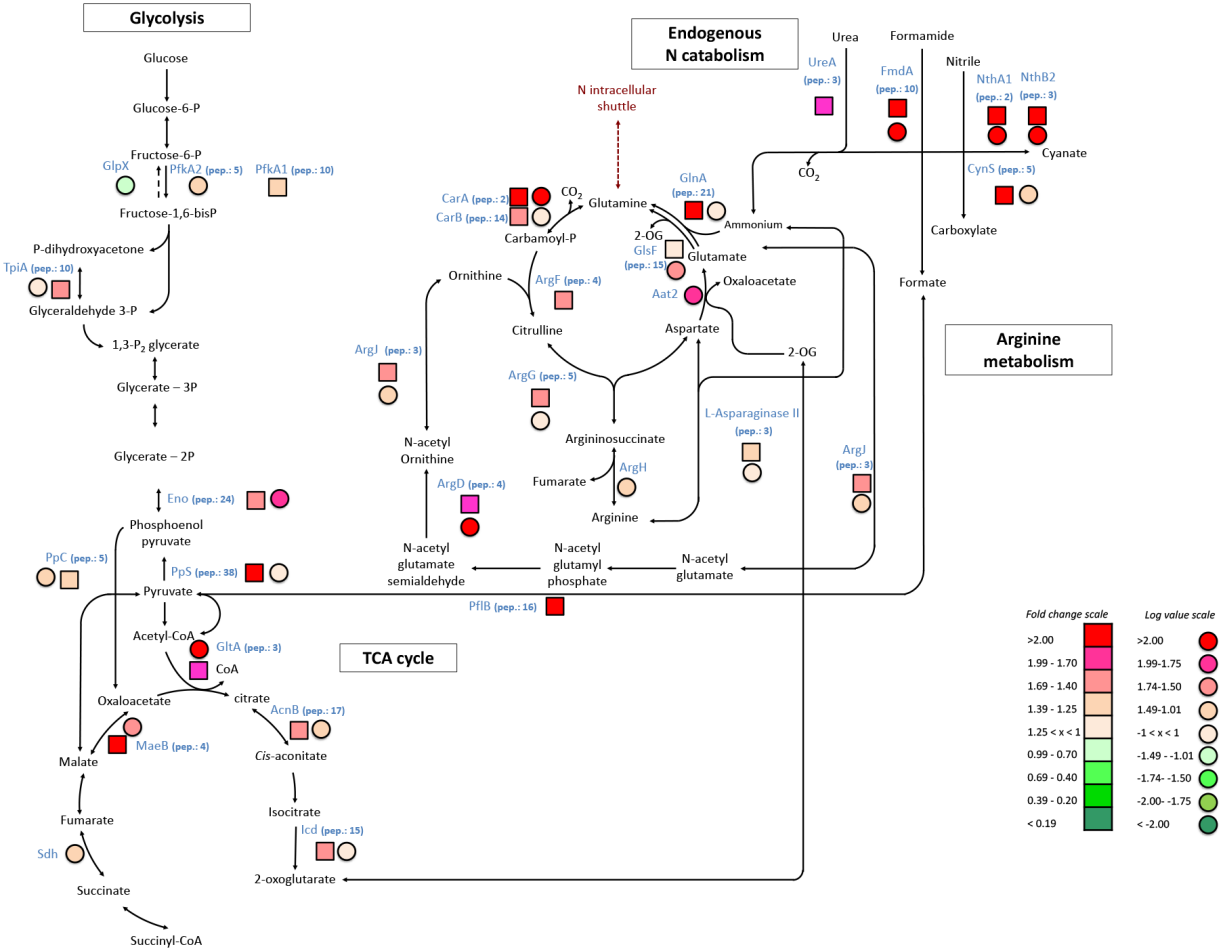
The molecular response of *Arthrospira* sp. PCC 8005 to nitrogen limitation: the glycolysis, endogenous-N and arginine metabolism. Molecular pathways were predicted according to the Genoscope and KEGG databases. The squares correspond to protein abundance (nitrogen limitation versus control) and the circles correspond to mRNA expression (nitrogen limitation versus control). The data presented here were taken from three biologically independent replicates.

Proteomics as well as transcriptomics indicated that *Arthrospira* activated its N acquisition mechanisms in response to the N depletion in the medium. An up-regulation of membrane transporters for uptake of nitrate (NrtA) or alternative N sources (amino acids, AapJ and LivJ; putrescine, PotG) was observed at protein level (Table 2). Enzymes involved in degradation of organic N sources seemed to be enhanced, α-subunit of urease (UreA), formamidase (FmdA) and cyanase (CynS) were up-regulated at protein level and/or mRNA level (Table 2 and Fig 2). The up-regulation of these genes was also shown in N-limited *Synechocystis* sp. PCC 6803 [27]. The nitrile hydratase gene cluster (NthA1 and NthB2) was also up-regulated. This gene cluster allows this strain to utilize nitriles as a N source. This gene cluster is uncommon in cyanobacteria and is unique to *Arthrospira* PCC 8005 among the sequenced *Arthrospira* strains [28]. Since these alternative N sources were not present in the medium, however, these strategies could probably not balance the depletion of N from the medium.

**Table 2:** Quantitative transcriptomics with differential expression (Log-value) and proteomics with number of peptides (# Pept), differential abundance (Fold change) in three biological replicates with their associated *p*-value.

			TRANSCRIPTOMICS		PROTEOMICS		
Accession number	Abbreviation	Gene/proteins name	*p*-value	Log value	# Pept	*p*-value	Fold change
***Nitrogen acquisition mechanisms***							
ARTHROv5_40621	*nrtA*	ABC Nitrate transport system. periplasmic component NrtA	0.44	0.61	13	0.00	1.66
ARTHROv5_10389	*aapJ*	General L-amino acid-binding periplasmic protein AapJ	0.11	0.27	17	0.00	2.60
ARTHROv5_10488		putative ABC-type branched-chain amino acid transport systems. periplasmic component. LivJ-like	0.06	0.87	14	0.00	2.58
ARTHROv5_60400	*potG*	putrescine transporter subunit: ATP-binding component of ABC superfamily	0.01	0.49	3	0.00	2.70
ARTHROv5_30069	*ureA*	Urease subunit gamma (Urea amidohydrolase subunit gamma)	0.30	-0.18	3	0.02	1.88
ARTHROv5_20218	*fmdA*	formamidase (formamide amidohydrolase)	0.00	2.16	10	0.00	2.53
ARTHROv5_11880	*cynS*	cyanase	0.00	1.25	5	0.03	2.94
ARTHROv5_60175	*nthA1*	Nitrile hydratase alpha subunit	0.00	2.13	2	0.00	3.23
ARTHROv5_60176	*nthB2*	Nitrile hydratase beta subunit	0.00	2.32	3	0.00	2.61
ARTHROv5_50271	*glnB*	protein P-II	0.00	1.72	7	0.01	2.00
ARTHROv5_30796	*ntcB*	nitrogen assimilation transcriptional activator (LysR family)	0.00	5.47			
***De novo protein synthesis***							
ARTHROv5_20177	*rpsO*	30S ribosomal subunit protein S15	0.03	-0.36	4	0.28	1.40
ARTHROv5_11037	*rpsC*	30S ribosomal subunit protein S3	0.00	-1.27	3	0.34	1.06
ARTHROv5_40190	*rpsD*	30S ribosomal subunit protein S4	0.00	-1.01	8	0.39	1.19
ARTHROv5_50072	*rpsG*	30S ribosomal subunit protein S7	0.04	-0.33	9	0.00	1.42
ARTHROv5_11044	*rpsH*	30S ribosomal subunit protein S8	0.00	-1.48	3	0.71	1.03
ARTHROv5_11058	*rpsI*	30S ribosomal subunit protein S9	0.00	-1.53	5	0.22	0.82
ARTHROv5_11035	*rpsS*	30S ribosomal subunit protein S19	0.00	-1.81	1	0.49	1.08
***Degradation proteins and recycle internal N reserves***							
ARTHROv5_11717	*pepA*	cytosol aminopeptidase	0.01	-0.54	7	0.03	1.41
ARTHROv5_40288		Alpha/beta hydrolase fold protein	0.17	-0.66	5	0.01	1.30
ARTHROv5_60975	*ybbK*	protease, membrane anchored, stomatin/prohibitin homologs	0.10	0.22	16	0.04	1.36
ARTHROv5_30200		Peptidase C14, caspase catalytic subunit p20	0.00	1.46	3	0.00	1.47
ARTHROv5_30650	*ilvH*	Acetolactate synthase small subunit	0.28	-0.15	3	0.03	1.89
ARTHROv5_10603	*ilvD*	Dihydroxy-acid dehydratase	0.04	-0.38	8	0.04	1.35
ARTHROv5_11016	*ilvY*	Acetolactate synthase	0.00	1.54	7	0.01	1.50
ARTHROv5_60603	*metE*	5-methyltetrahydropteroyltriglutamate—homocyste ine methyltransferase	0.01	-0.75	24	0.00	1.43
ARTHROv5_60705	*trpD*	Anthranilate phosphoribosyltransferase	0.00	0.99	5	0.00	1.85
ARTHROv5_10309	*cysk3*	Cysteine synthase	0.00	0.96	5	0.00	1.52
ARTHROv5_10293	*ppc*	Phosphoenolpyruvate carboxylase	0.00	1.47	5	0.00	1.34
ARTHROv5_10688	*gltA*	citrate synthase	0.00	2.92	3	0.02	1.83
ARTHROv5_11363	*icd*	Isocitrate dehydrogenase [NADP]	0.00	0.88	15	0.01	1.63
ARTHROv5_12133	*glnA*	glutamine synthetase	0.00	0.92	21	0.00	2.09
ARTHROv5_50078	*glsF*	Ferredoxin-dependent glutamate synthase. large subunit	0.00	1.52	15	0.02	1.23
ARTHROv5_30789	*aat2*	Aspartate aminotransferase	0.00	1.80	4	0.20	1.68
ARTHROv5_61056	*nblA*	Phycobilisome degradation protein	0.00	3.35			
ARTHROv5_11397	*nblB2*	Phycocyanin alpha phycocyanobilin lyase related protein	0.00	-1.08	5	0.40	0.98
***Glycogen synthesis***							
ARTHROv5_60834	*glgB*	1.4-alpha-glucan branching enzyme	0.01	-0.47	7	0.01	1.37
ARTHROv5_11073	*glgC*	glucose-1-phosphate adenylyltransferase	0.00	-0.72	16	0.01	1.55
ARTHROv5_20114	*glgP*	Glycogen/starch/alpha-glucan phosphorylase	0.00	-1.35			
***Gluconeogenesis***							
ARTHROv5_10771	*glpX*	D-fructose 1.6-bisphosphatase class II	0.00	-1.38	27	0.25	0.91
ARTHROv5_11360	*pck*	phosphoenolpyruvate carboxykinase	0.02	0.70			
ARTHROv5_40254	*sdhB*	succinate dehydrogenase iron-sulfur subunit	0.00	1.49	7	0.18	1.25
ARTHROv5_61117	*maeB*	Malate dehydrogenase (oxaloacetate-decarboxylating) (NADP(+))	0.00	1.63	4	0.00	3.34
ARTHROv5_10436	*pps*	phosphoenolpyruvate synthase	0.00	0.86	38	0.00	3.47
ARTHROv5_11119	*eno*	enolase	0.00	1.81	24	0.00	1.65
***Lipid and polyhydroxybutyrate metabolism***							
**ARTHROv5_30343**	*accA*	Acetylcoenzyme A carboxylase	0.01	-0.40	2	0.22	1.49
**ARTHROv5_30176**	*fabD*	Malonyl CoA-acyl carrier protein transacylase	0.02	-1.00			
**ARTHROv5_30177**	*fabH*	3-oxoacyl-[acyl-carrier-protein] synthase 3	0.02	-0.98			
**ARTHROv5_10239**	*acpP*	acyl carrier protein	0.00	-1.03	5	0.01	0.66
**ARTHROv5_50103**	*acsA*	Acetyl-coenzyme A synthetase	0.01	-0.72	13	0.01	0.72
**ARTHROv5_30178**	*plsX*	Fatty acid/phospholipid synthesis protein	0.03	-1.05			
**ARTHROv5_10500**	*phaC*	Poly(R)-hydroxyalkanoic acid synthase, class III, PhaC subunit	0.23	-0.25	2	0.47	1.19
**ARTHROv5_10499**	*phaE*	Poly(R)-hydroxyalkanoic acid synthase, class III, PhaE subunit	0.01	-0.55	1	0.73	0.92
**ARTHROv5_60059**	*phbA*	acetyl-CoA acetyltransferase with thiolase domain (Acetoacetyl-CoA thiolase)	0.01	1.18	6	0.06	1.31
***Photosynthesis***							
ARTHROv5_11553	*cpcB*	C-phycocyanin beta subunit	0.00	-1.65	41	0.02	0.66
ARTHROv5_11555	*cpcC1*	Phycobilisome 32 kDa linker polypeptide. phycocyanin-associated. rod 1	0.00	-3.83	15	0.00	0.11
ARTHROv5_11556	*cpcC2*	Phycobilisome 32 kDa linker polypeptide. phycocyanin-associated. rod 2	0.00	-2.34	34	0.02	0.69
ARTHROv5_11557	*cpcD*	Phycobilisome 8.9 kDa linker polypeptide. phycocyanin-associated. rod (Rod-capping linker protein)	0.01	-1.36	11	0.52	0.76
ARTHROv5_40726	*cpcG*	phycobilisome rod-core linker protein	0.00	-1.11	28	0.56	1.10
ARTHROv5_10637	*apcA*	Allophycocyanin alpha subunit	0.00	-1.42	25	0.09	1.30
ARTHROv5_10636	*apcB*	Allophycocyanin beta subunit	0.00	-2.41	27	0.46	0.79
ARTHROv5_10635	*apcC*	Phycobilisome 7.8 kDa linker polypeptide. allophycocyanin-associated. core (LC 7.8)	0.00	-1.94	7	0.19	1.16
ARTHROv5_12132	*apcF*	allophycocyanin beta-18 subunit	0.02	-1.04	16	0.54	1.00
ARTHROv5_11660	*hemF*	coproporphyrinogen III oxidase	0.99	0.00	9	0.00	0.61
ARTHROv5_10770	*hemA*	glutamyl tRNA reductase (GluTR)	0.00	-2.05	1	0.98	1.04
ARTHROv5_61056	*nblA*	Phycobilisome degradation protein	0.00	3.35			
ARTHROv5_10235	*psaC*	Photosystem I iron-sulfur center (Photosystem I subunit VII) (9 kDa polypeptide) (PSI-C) (PsaC)	0.10	0.42	15	0.11	1.38
ARTHROv5_30080	*psaD*	Photosystem I reaction center subunit II (Photosystem I 16 kDa polypeptide) (PSI-D)	0.92	-0.01	20	0.02	1.44
ARTHROv5_30656	*psaJ*	Photosystem I reaction center subunit IX	0.00	-1.07	/	/	/
ARTHROv5_50157	*psaL*	Photosystem I reaction center subunit XI (PSI-L) (PSI subunit V)	0.00	-1.30	2	0.66	1.12
ARTHROv5_60554	*psbF*	Cytochrome b559 subunit beta (PSII reaction center subunit VI)	0.00	-1.53			
ARTHROv5_30303	*psbI*	Photosystem II reaction center protein I	0.00	-1.20			
ARTHROv5_40153	*psbO*	photosystem II manganese-stabilizing polypeptide precursor (MSP)	0.00	-3.10	6	0.72	1.05
ARTHROv5_40969	*psbU*	Photosystem II 12 kDa extrinsic protein precursor (PS II complex 12 kDa extrinsic protein) (PSII-U)	0.00	-1.28	6	0.41	1.22
ARTHROv5_11112	*psb28*	Photosystem II reaction center psb28 protein (Photosystem II reaction center W protein) (Photosystem II 13 kDa protein)	0.73	0.07	12	0.28	1.30
ARTHROv5_50094	*psbV*	Cytochrome c-550 precursor (Cytochrome c550) (Low-potential cytochrome c)	0.00	-2.36	11	0.23	1.38
ARTHROv5_60535	*atpH*	ATP synthase delta chain; ATP synthase F1. delta subunit	0.00	-3.19	9	0.17	0.90
ARTHROv5_60534	*atpF*	ATP synthase B chain (Subunit I)	0.00	-4.00	7	0.17	0.74
ARTHROv5_60533	*atpG2*	ATP synthase B' chain (Subunit II)	0.00	-3.97	2	0.12	1.47
ARTHROv5_60536	*atpA*	F1 sector of membrane-bound ATP synthase. alpha subunit	0.00	-2.31	20	0.10	0.92
ARTHROv5_60531	*atpB*	ATP synthase a chain (ATPase protein 6)	0.00	-2.39			
ARTHROv5_60532	*atpE*	ATP synthase subunit C. membrane-bound. F0 sector; DCCD-binding	0.00	-2.32			
ARTHROv5_60530	*atpI*	ATP synthase protein I	0.00	-2.08			
ARTHROv5_50351	*cbbL*	Ribulose bisphosphate carboxylase large chain (RuBisCO large subunit)	0.00	-1.12	57	0.10	0.83
ARTHROv5_50349	*cbbS*	Ribulose bisphosphate carboxylase small chain	0.00	-1.82	15	0.13	0.86
ARTHROv5_10771	*glpX*	D-fructose 1.6-bisphosphatase class II	0.00	-1.38	27	0.25	0.91

The main regulators of the N metabolism are the transcription factor NtcA, which controls the expression of genes involved in the N metabolism, and the signaling protein PII (encoded by *glnB*), which fine-tunes the cellular metabolism in response to fluctuating intracellular C/N ratios [53]. The *glnB* gene is transcriptionally activated by NtcA and the *glnB* product, the protein PII, is required for the activation of NtcA-regulated genes [22]. The activity of the PII protein itself, depends on its phosphorylation by binding 2-OG and ATP [54]. In our study, PII was up-regulated at the protein and transcript levels. However, NtcAwas not up- or down-regulated. The up-regulation of the *glnB* gene under N-stress without a change in expression of NtcA was previously reported within *Synechocystis* sp. PCC 6803 [27].

In addition, mRNA of *NtcB*, a nitrate assimilation transcriptional activator, was more abundant under N-depletion, and might indicate a positive activation of the use of alternative N-sources reported in Table 2. Similar responses to N limitation have already been reported in a previous proteomic analysis of *Arthrospira* [14] as well as in other cyanobacteria [25].

### 4. Down-regulation of *de novo* protein synthesis

Transcriptomic data indicated that exhaustion of N from the medium resulted in down-regulation of *de novo* protein synthesis. Transcripts of many ribosomal genes were down-regulated. No change in ribosomal proteins was observed in the proteomic analysis, suggesting that *Arthrospira* might maintain its existing ribosomes but stops creating new ones. The observed down-regulation of protein synthesis was reflected in the decrease in total protein content of the N-limited *Arthrospira* (see above). A down-regulation of ribosomal gene transcription under N limitation has also been observed in *Synechocystis* sp. PCC 6803 [26,27].

### 5. Degradation of proteins and recycling of N

**Fig 3:**
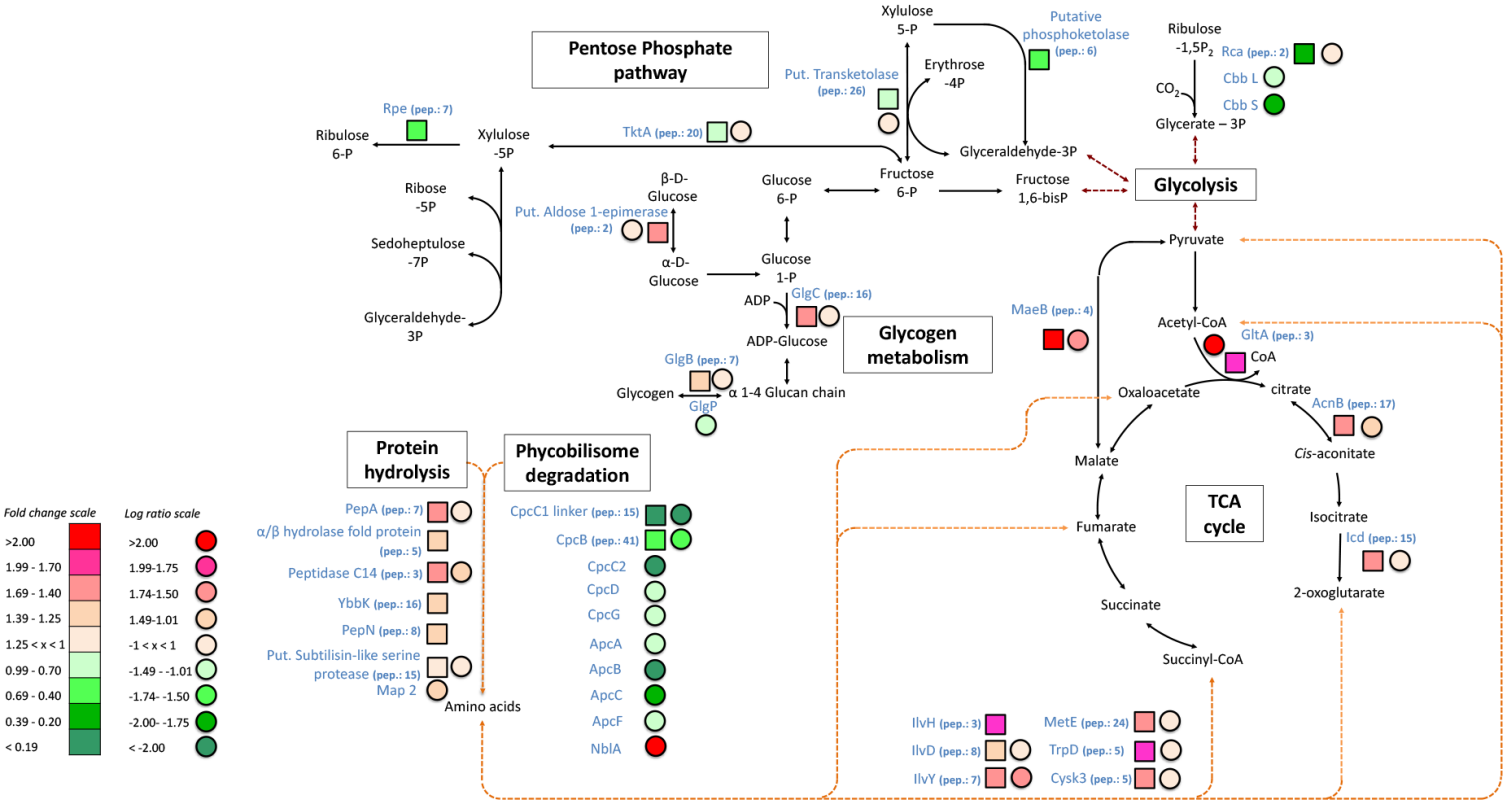
The molecular response of *Arthrospira* sp. PCC 8005 to nitrogen limitation: the pentose phosphate pathway (OPP), protein and phycobilisome degradation, glycogen metabolism and citric acid cycle. Molecular pathways were predicted according to the Genoscope and KEGG databases. The squares correspond to protein abundance (nitrogen limitation versus control) and the circles correspond to mRNA expression (nitrogen limitation versus control). The data presented here were taken from four biologically independent replicates.

Transcriptomic and proteomic information suggest that N-limited *Arthrospira* actively degrades its proteins and recycles the N atoms associated with the amino acid residues. Indeed, some proteases were up-regulated at gene and/or protein level (*e*.*g*., Peptidase C14, PepA, Ybkk…) (Table 2 and Fig 3). In cyanobacteria, particularly in non-diazotrophic species, the phycobilisome proteins are considered as an important N-storage reservoir and are actively degraded under N-starvation, as has been previously reported in studies of other cyanobacteria[26,27,54]. The *nblA* gene encoding the phycobilisome degradation protein was found to be up-regulated at mRNA level, indicating that *Arthrospira* sp. PCC 8005 might indeed degrade its phycobilisomes to supply N for other metabolic processes. Although the NblA protein did not show an increased abundance, the degradation of the phycobilisome could be in agreement with the observed reduction in phycocyanin content (Table 1).

In addition, transcriptomic analyses showed a reduced expression of phycocyanin genes (*apcA*, *apcB*, *apcC* and *apcF*). Phycobilisome linker proteins (CpcC1, CpcC2 and CpcD) were also down-regulated at transcript as well as protein level (Table 2).

There are indications that the N associated with amino acids derived from protein degradation are recycled to provide a source of intracellular N. The amine group of amino acids is transferred to 2-oxoglutarate (2-OG) by glutamine synthetase (GlnA) and glutamate synthase (GlsF), which were both significantly up-regulated at the proteomic and transcriptomic level (Table 2 and Fig 2). Up-regulation of GlnA and GlsF in response to N limitation has been previously observed in *Arthrospira* PCC 8005 and *Synechocystis* sp. PCC 6803 [14,25,27]. 2-OG required for accepting amine groups is provided through the TCA cycle enzymes phosphoenolpyruvate carboxylase (*ppc*), citrate synthase (*gltA*) and isocitrate dehydrogenase (*icd*), which were up-regulated at the transcriptomic and proteomic level (Fig 2). This stimulation of amino acid biosynthesis and TCA cycle intermediates under N deprivation has also been previously observed in *Arthrospira platensis* [56] and in *Synechocystis* sp. PCC 6803. [27] under N limitation. The fact that arginine synthesis genes (*argF*, *argG* and ArgH) as well as the cyanophycin synthetase gene *cphA* were up-regulated at the protein or mRNA level might indicate that internal N is temporarily stored in cyanophycin granules. However, this is in contradiction to previous studies of N-limited *Synechocystis* sp. PCC 6803 and *Arthrospira* sp. PCC 8005, where cyanophycin was degraded under N-limitation [14,35]. Cyanophycin is an internal N storage reserve in cyanobacteria and is known to serve as a transient N reservoir in N-starved cyanobacteria [57]. It seems that the regulation of cyanophycin synthesis depends on a complex interrelation between cyanophycin synthesis, the arginine catabolism and photosynthesis [55]. Amino acid biosynthesis genes were found to be up-regulated at transcriptional level, *e*.*g*., *ilvH*, *metE* and *trpD* (Table 2 and Fig 3), suggesting that this internal N reserves are used for production of new proteins. All this evidence suggests that N-limited *Arthrospira* degrades non-essential proteins to mobilize N for production of new proteins that are essential to maintain its activity in a low N medium.

### 6. Conversion of proteins into carbohydrates through gluconeogenesis

A recent study on dynamic metabolic profiling of the cyanobacterium *Arthrospira platensis* under N limitation suggested that a part of the accumulated glycogen under N-limiting conditions is derived from protein C through gluconeogenesis in *Arthrospira platensis* sp. PCC 8005 [14,56]. Although some key enzymes in the gluconeogenesis (phosphoenolpyruvate carboxykinase (Pck), fructose 1.6 bisphosphatase (GlpX) and glucose 6 phosphatase) were not up-regulated under N limitation, the succinate dehydrogenase gene (*sdh*) and phosphoenolpyruvate synthase protein (PpS) showed a higher abundance. Moreover malate dehydrogenase (MaeB) and enolase (Eno) were up-regulated at both transcriptional and protein level, all evidencing a stimulation of the gluconeogenesis (Table 2 and Fig 2).

Although a part of C-glycogen stock might be derived from protein C through gluconeogenesis, an analysis of changes in the productivity and composition of the biomass during N limitation suggested that the contribution of protein C for glycogen production in N-limited *Arthrospira* cells is likely limited. Prior to N depletion, the biomass concentration was about 0.3 g L^-1^, with 0.11 g L^-1^ as proteins (36%) and 0.04 g L^-1^ as carbohydrates (13%) (Table 1). When N was depleted from the medium, the biomass concentration in the culture approximately doubled to 0.6 g L^-1^. Due the changes in biomass composition, the protein content of the culture dropped to 0.07 g L^-1^ (10.4%), while the carbohydrate content increased to 0.44 g L^-1^ (73.7%). This represented a loss of 0.04 g L^-1^ protein and a production of 0.40 g L^-1^ carbohydrates. From this calculation it is clear that protein C was not the main contributor to glycogen production under N-limited conditions. Hence, C for glycogen production was most likely provided by residual photosynthetic carbon fixation.

### 7. Up-regulation of glycogen synthesis

In agreement with the massive increase in carbohydrate concentration in the biomass of the N-limited treatment, an up-regulation of the glycogen synthesis metabolism was observed. Glycogen synthesis proteins like 1,4-alpha-glucan branching enzyme (GlgB) and glucose-1-phosphate adenylyltransferase (GlgC) were up-regulated, and the glycogen degradation gene glycogen/starch/alpha-glucan phosphorylase (glgP) was down-regulated (Table 2 and Fig 3). In [25], they reported not only an up-regulation of *glgB* but also of *glgP* during accumulation of glycogen in N-limited *Synechocystis* sp. PCC 6803. In this study, no up-regulation of glycogen synthase was observed. The lack of response of glycogen synthase has also been observed in N-limited *Synechocystis* sp. PCC 6803 [55]. This might suggest that the glycogen production is controlled by the supply of ADP-glucose rather than by the amount of the enzyme [55].

### 8. Changes in lipid and polyhydroxybutyrate metabolism

In most eukaryotic microalgae, lipids are accumulated under N-limitation [6,58,59]. In cyanobacteria, the accumulation of lipids under N limitation has been observed in some species (e.g. *Oscillatoria*), but not in *Arthrospira* [6]. Phosphoenolpyruvate is the substrate for the synthesis of fatty acids (via acetylcoenzyme A) [18]. Although the conversion of phosphoenolpyruvate to oxaloacetate by *ppc* was up-regulated (as mentioned above), acetylcoenzyme A carboxylase (*accA*), which catalyses the rate-limiting step in the fatty acid biosynthesis was not up-or down-regulated. Two other important enzymes for the fatty acid biosynthesis, malonyl CoA-acyl carrier protein transacylase (*fabD*) and 3-oxoacyl-[acyl-carrier-protein] synthase 3 (*fab H*) were both down-regulated. Also the gene encoding the acyl carrier protein (*acpP*) and the acetylcoenzyme A synthetase protein (AcsA) were down-regulated. In [26], they observed a down-regulation of *accA* and *acpP* at transcript level in *Synechocystis* sp. PCC 6803 under N-limitation, although these genes were up-regulated at protein level. The first step in the lipid biosynthesis is the formation of 1-acyl-sn-glycerol-3-phosphate and is catalyzed by the *pls X* gene encoding the fatty acid/phospholipid synthesis protein. This gene was also down-regulated in our study. And finally, a down-regulation of the oxidative pentose phosphate (OPP) pathway, which is the major source of reducing power in cyanobacteria, was observed (Fig 3) [60]. These findings all suggest that the fatty acid biosynthesis is down-regulated and is in agreement with the observed reduced fatty acid methyl ester content in N-limited *Arthrospira* (Table 1). Because a significant proportion of the fatty acids is associated with photosynthetic thylakoid membranes, the reduction in fatty acid content may be result of a down-regulation of photosynthesis (see below).

Cyanobacteria are also known to accumulate polyhydroxybutyrate as carbon and energy storage product [18]. High concentrations of polyhydroxybutyrate have been reported from *Arthrospira* sp. (about 6%) [61]. In *Synechocystis sp*. PCC 6803, the polyhydroxybutyrate content increased from 2.4% to 13.5% in response to N limitation [62]. As for lipids, the substrate for synthesis of polyhydroxybutyrate is acetylcoenzyme A. The enzymes poly(R)-hydroxyalkanoic acid synthase subunit C (*phaC*) and subunit E (*phaE*) catalyse the final stages in the polyhydroxybutyrate synthesis [62,63]. We observed no up- or down-regulation of *phaC* and *phaE* under N-limitation. However, β-ketothiolase (*phbA*), which catalyses the first step of the PHB synthesis was significantly up-regulated.

It may be interesting to include polyhydroxybutyrate measurement in future studies to know if *Arthrospira* accumulates polyhydroxybutyrate under N-stress.

### 9. Down-regulation of photosynthesis

It is evident from the transcriptomic and proteomic data that N-limited *Arthrospira* had down-regulated its photosynthesis. In agreement with the observed decrease in chlorophyll concentration in the biomass, we observed a lower expression of chlorophyll synthesis genes (*hem*). Chlorosis caused by N stress was previously shown in *Arthrospira* sp. PCC 8005 [14] and in *Synechocystis* sp. PCC 6803 [27].

The photosystem I (*psa*) and II (*psb*) genes and ATP synthesis (*atp*) genes were all down-regulated, but the abundance of the corresponding proteins seemed to remain unchanged (Table 2). This suggests that no new photosynthetic proteins are formed under N limitation but that existing photosynthetic proteins are not actively being degraded, as has been observed in *Synechocystis* sp. PCC 6803 [26,35]. Under N limitation, photosynthetic carbon acquisition was down-regulated at the level of light harvesting and CO_2_ fixation. Indeed, the key enzyme ribulose-1,5-bisphoshate carboxylase/oxygenase RuBisCO (*cbbL* and *cbbS*) exhibits a lower transcript level and the regulator ribulose bisphosphate carboxylase/oxygenase activase (Rca) a lower abundance of proteins (Table 2 and Fig 3). Also, the transcription of fructose 1.6-bisphosphatase (*glpX*), which is a key enzyme for the photosynthetic CO_2_ assimilation, was down-regulated (Table 2 and Fig 2). Similar results have already been reported for *Synechocystis* sp. PCC 6803 [25] and *Arthrospira* PCC 8005 [14].

If carbon acquisition is clearly down-regulated in N-limited *Arthrospira* cells, why were the growth rate and final biomass concentration not reduced by N limitation? Our data suggest that in the N-limited culture, most C fixed during photosynthesis was converted into glycogen, while in the control culture a large part of the C was used to produce proteins. Production of carbohydrates requires much less energy than production of proteins. Only 0.012 g ATP is required to produce 1 g of storage carbohydrates as opposed to 0.082 g ATP for the production of proteins (when nitrate is used as a N source) [64]. This corresponds to 1.09 g glucose equivalents for the production of glycogen as opposed to 2.45 g glucose equivalents for production of proteins [65]. Thus, *Arthrospira* is capable of producing new biomass at a similar rate in N-limited as in N-replete conditions because the energy produced during photosynthesis is converted into new biomass in a more efficient way in N-limited conditions (when mainly carbohydrates are produced) than in N-replete conditions (when mainly proteins are produced).

## Conclusion

N-limited *Arthrospira* cultures were able to produce biomass at the same rate as N-replete cultures up to 5 days after N was fully depleted from the medium. In N-limited conditions, *de novo* protein synthesis was down-regulated, and existing proteins were partly converted into carbohydrates and the N-containing group was internally recycled, respectively through gluconeogenesis and TCA cycle. Photosynthetic energy production and carbon fixation were down-regulated, which resulted in a reduced availability of energy to the cells. The glycogen synthesis was up-regulated suggesting that photosynthetic energy was channeled towards glycogen production rather than protein production.

The glycogen synthesis requires lower energy demand than protein synthesis. This might explain why the N-limited *Arthrospira* cultures were able to achieve an equally high biomass production rate as the control cultures, despite a weaker activity of photosynthetic energy production.

## Supporting Information

S1 Table List of genes showing changes in expression between nitrogen limited and control conditions. Data presented here are issued from three independent biological replicates.
(XLSX)

S2 Table List of proteins showing significant changes of abundance level (*p*-value < 0.05) between nitrogen limited and control conditions. The fold changes are the ratios of the abundance of proteins between nitrogen limited and control. Data presented here are issued from three independent biological replicates.
(XLSX)

## References

[1] Borowitzka MA, Moheimani NR. Sustainable biofuels from algae. Mitig Adapt Strateg Glob Chang. 2013;18: 13–25. 10.1007/s11027-010-9271-9

[2] Pienkos PT, Darzins A. The promise and challenges of of microalgal-derived biofuels. Biofuels, Bioprod biorefining. 2009;3: 431–440. 10.1002/bbb

[3] Lam MK, Lee KT. Microalgae biofuels: A critical review of issues, problems and the way forward. Biotechnol Adv. Elsevier Inc.; 2012;30: 673–90. 10.1016/j.biotechadv.2011.11.008 22166620

[4] Subramanian S, Barry AN, Pieris S, Sayre RT. Comparative energetics and kinetics of autotrophic lipid and starch metabolism in chlorophytic microalgae: implications for biomass and biofuel production. Biotechnol Biofuels. Biotechnology for Biofuels; 2013;6: 150 10.1186/1754-6834-6-150 24139286 PMC4015678

[5] Benemann J. Microalgae for biofuels and animal feeds. Energies. 2013;6: 5869–5886. 10.3390/en6115869

[6] Griffiths MJ, Harrison STL. Lipid productivity as a key characteristic for choosing algal species for biodiesel production. J Appl Phycol. 2009;21: 493–507. 10.1007/s10811-008-9392-7

[7] De Philippis R, Sili C, Vincenzini M. Glycogen and poly-hydroxybutyrate synthesis in Spirulina maxima. J Gen Microbiol. 1992;138: 1623–1628. 10.1099/00221287-138-8-1623

[8] Gordillo FJL, Jim C, Figueroa L, Niell FX. Effects of increased atmospheric CO2 and N supply on photosynthesis, growth and cell composition of the cyanobacterium Spirulina platensis (Arthrospira). J Appl Phycol. 1999;10: 461–469.

[9] Sassano CEN, Gioielli LA, Ferreira LS, Rodrigues MS, Sato S, Converti A, et al Evaluation of the composition of continuously-cultivated Arthrospira (Spirulina) platensis using ammonium chloride as nitrogen source. Biomass and Bioenergy. Elsevier Ltd; 2010;34: 1732–1738. 10.1016/j.biombioe.2010.07.002

[10] Aikawa S, Izumi Y, Matsuda F, Hasunuma T, Chang J-S, Kondo A. Synergistic enhancement of glycogen production in Arthrospira platensis by optimization of light intensity and nitrate supply. Bioresour Technol. Elsevier Ltd; 2012;108: 211–5. 10.1016/j.biortech.2012.01.004 22277210

[11] Markou G, Chatzipavlidis I, Georgakakis D. Effects of phosphorus concentration and light intensity on the biomass composition of Arthrospira (Spirulina) platensis. World J Microbiol Biotechnol. 2012;28: 2661–2670. 10.1007/s11274-012-1076-4 22806192

[12] Depraetere O, Pierre G, Deschoenmaeker F, Badri H, Foubert I, Leys N, et al Harvesting carbohydrate-rich Arthrospira platensis by spontaneous settling. Bioresour Technol. Elsevier Ltd; 2014;180: 16–21. 10.1016/j.biortech.2014.12.084 25585253

[13] Becker EW. Micro-algae as a source of protein. Biotechnol Adv. 2007;25: 207–10. 10.1016/j.biotechadv.2006.11.002 17196357

[14] Deschoenmaeker F, Facchini R, Leroy B, Badri H, C Z, Wattiez R. Proteomic and cellular views of Arthrospira sp. PCC 8005 adaptation to nitrogen depletion. Microbiology. 2014;160: 1224–1236. 10.1099/mic.0.074641-0 24648480

[15] González-Fernández C, Ballesteros M. Linking microalgae and cyanobacteria culture conditions and key-enzymes for carbohydrate accumulation. Biotechnol Adv. 2012;30: 1655–1661. 10.1016/j.biotechadv.2012.07.003 22820270

[16] Ball SG. Strains, media, growth and incubation conditions. Plant Sci. 1990;66: 1–9.

[17] Brányiková I, Maršálková B, Doucha J, Brányik T, Bišová K, Zachleder V, et al Microalgae—novel highly efficient starch producers. Biotechnol Bioeng. 2011;108: 766–76. 10.1002/bit.23016 21404251

[18] Quintana N, Van der Kooy F, Van de Rhee MD, Voshol GP, Verpoorte R. Renewable energy from cyanobacteria: energy production optimization by metabolic pathway engineering. Appl Microbiol Biotechnol. 2011;91: 471–90. 10.1007/s00253-011-3394-0 21691792 PMC3136707

[19] Radakovits R, Jinkerson RE, Darzins A, Posewitz MC. Genetic engineering of algae for enhanced biofuel production. Eukaryot Cell. 2010;9: 486–501. 10.1128/EC.00364-09 20139239 PMC2863401

[20] Reijnders MJMF, van Heck RGA, Lam CMC, Scaife MA, Martins dos Santos VAP, Smith AG, et al Green genes: bioinformatics and systems-biology innovations drive algal biotechnology. Trends Biotechnol. Elsevier Ltd; 2014;32: 617–627. 10.1016/j.tibtech.2014.10.003 25457388

[21] Sauer J, Schreiber U, Schmid R, Völker U, Forchhammer K. Nitrogen starvation-induced chlorosis in Synechococcus PCC 7942. Low-level photosynthesis as a mechanism of long-term survival. Plant Physiol. 2001;126: 233–43. Available: http://www.pubmedcentral.nih.gov/articlerender.fcgi?artid=102297&tool=pmcentrez&rendertype=abstract 11351086 10.1104/pp.126.1.233 PMC102297

[22] Tolonen AC, Aach J, Lindell D, Johnson ZI, Rector T, Steen R, et al Global gene expression of Prochlorococcus ecotypes in response to changes in nitrogen availability. Mol Syst Biol. 2006;2: 53 10.1038/msb4100087 17016519 PMC1682016

[23] Harke MJ, Gobler CJ. Global transcriptional responses of the toxic cyanobacterium, Microcystis aeruginosa, to nitrogen stress, phosphorus stress, and growth on organic matter. PLoS One. 2013;8: e69834 10.1371/journal.pone.0069834 23894552 PMC3720943

[24] Ehira S, Ohmori M. NrrA, a nitrogen-responsive response regulator facilitates heterocyst development in the cyanobacterium Anabaena sp. strain PCC 7120. Mol Microbiol. 2006;59: 1692–703. 10.1111/j.1365-2958.2006.05049.x 16553876

[25] Osanai T, Imamura S, Asayama M, Shirai M, Suzuki I, Murata N, et al Nitrogen induction of sugar catabolic gene expression in Synechocystis sp. PCC 6803. DNA Res. 2006;13: 185–95. 10.1093/dnares/dsl010 17046957

[26] Huang S, Chen L, Te R, Qiao J, Wang J, Zhang W. Complementary iTRAQ proteomics and RNA-seq transcriptomics reveal multiple levels of regulation in response to nitrogen starvation in Synechocystis sp. PCC 6803. Mol Biosyst. 2013;9: 2565–74. 10.1039/c3mb70188c 23942477

[27] Krasikov V, Aguirre von Wobeser E, Dekker HL, Huisman J, Matthijs HCP. Time-series resolution of gradual nitrogen starvation and its impact on photosynthesis in the cyanobacterium Synechocystis PCC 6803. Physiol Plant. 2012;145: 426–439. 10.1111/j.1399-3054.2012.01585.x 22289076

[28] Janssen PJ, Morin N, Mergeay M, Leroy B, Wattiez R, Vallaeys T, et al Genome sequence of the edible cyanobacterium Arthrospira sp. PCC 8005. J Bacteriol. 2010;192: 2465–6. 10.1128/JB.00116-10 20233937 PMC2863490

[29] Fujisawa T, Narikawa R, Okamoto S, Ehira S, Yoshimura H, Suzuki I, et al Genomic structure of an economically important cyanobacterium, Arthrospira (Spirulina) platensis NIES-39. DNA Res. 2010;17: 85–103. 10.1093/dnares/dsq004 20203057 PMC2853384

[30] Cheevadhanarak S, Paithoonrangsarid K, Prommeenate P, Kaewngam W, Musigkain A, Tragoonrung S, et al Draft genome sequence of Arthrospira platensis C1 (PCC9438). Stand Genomic Sci. 2012;6: 43–53. 10.4056/sigs.2525955 22675597 PMC3368399

[31] Lefort F, Calmin G, Crovadore J, Falquet J, Hurni J, Osteras M, et al Whole-genome shotgun sequence of Arthrospira platensis strain paraca, a cultivated andedible cyanobacterium. Genome Announc. 2014;2: 4–5. 10.1128/genomeA.00751-14.Copyright PMC4125771 25103760

[32] Matallana-Surget S, Derock J, Leroy B, Badri H, Deschoenmaeker F, Wattiez R. Proteome-wide analysis and diel proteomic profiling of the cyanobacterium Arthrospira platensis PCC 8005. PLoS One. 2014;9: e99076 10.1371/journal.pone.0099076 24914774 PMC4051694

[33] Huili W, Xiaokai Z, Meili L, Dahlgren R a, Wei C, Jaiopeng Z, et al Proteomic analysis and qRT-PCR verification of temperature response to Arthrospira (Spirulina) platensis. PLoS One. 2013;8: e83485 10.1371/journal.pone.0083485 24349519 PMC3861494

[34] Wang H, Yang Y, Chen W, Ding L, Li P, Zhao X, et al Identification of differentially expressed proteins of Arthrospira (Spirulina) platensis-YZ under salt-stress conditions by proteomics and qRT-PCR analysis. Proteome Sci. Proteome Science; 2013;11: 6 10.1186/1477-5956-11-6 23363438 PMC3599948

[35] Wegener KM, Singh AK, Jacobs JM, Elvitigala T, Welsh E a, Keren N, et al Global proteomics reveal an atypical strategy for carbon/nitrogen assimilation by a cyanobacterium under diverse environmental perturbations. Mol Cell Proteomics. 2010;9: 2678–89. 10.1074/mcp.M110.000109 20858728 PMC3101856

[36] Cogne G, Lehmann B, Dussap C-G, Gros J-B. Uptake of macrominerals and trace elements by the cyanobacterium Spirulina platensis (Arthrospira platensis PCC 8005) under photoautotrophic conditions: culture medium optimization. Biotechnol Bioeng. 2003;81: 588–93. 10.1002/bit.10504 12514808

[37] Vanveldhoven PP, Mannaerts GP. Inorganic and organic phosphate measurements in nanomolar range. Anal Biochem. 1987;161: 45–48. 3578786 10.1016/0003-2697(87)90649-x

[38] APHA. Standard methods for the examination of water and wastewater. 21st ed Eaton AD, Clesceri LS, Rice EW, Greenberg AE, editors. APHA, Washington D.C; 2005.

[39] Qualls RG. Determination of total nitrogen and phosphorus in water using persulfate oxidation: a modification for small sample volumes using the method of Koroleff (1983). University of Georgia 1989.

[40] Lichtenthaler HK, Buschmann C. Chlorophylls and carotenoids: measurement and characterization by UV-VIS spectroscopy Current protocols in food analytical chemistry. John Wiley & Sons, Inc; 2001 pp. F4.3.1–F4.3.8.

[41] Yoshikawa N, Belay A. Single-laboratory validation of a method for the determination of c-phycocyanin and allophycocyanin in Spirulina (Arthrospira) supplements and raw materials by spectrophotometry. J AOAC Int. 2008;91: 524–529. Available: http://www.ncbi.nlm.nih.gov/pubmed/18567296 18567296

[42] Nagase H, Yoshihara K, Eguchi K, Okamoto Y, Murasaki S, Yamashita R, et al Uptake pathway and continuous removal of nitric oxide from flue gas using microalgae. Biochem Eng J. 2001;7: 241–246. 10.1016/S1369-703X(00)00122-4

[43] DuBois M, Gilles K a., Hamilton JK, Rebers P a., Smith F. Colorimetric method for determination of sugars and related substances. Anal Chem. 1956;28: 350–356. 10.1021/ac60111a017

[44] Lepage G, Roy CC. Improved recovery of fatty acid through direct transesterification without prior extraction or purification. J Lipid Res. 1984;25: 1391–6. Available: http://www.ncbi.nlm.nih.gov/pubmed/6530596 6530596

[45] Cho S, Lee N, Park S, Yu J, Luong TT, Oh Y-K, et al Microalgae cultivation for bioenergy production using wastewaters from a municipal WWTP as nutritional sources. Bioresour Technol. 2013;131: 515–20. 10.1016/j.biortech.2012.12.176 23453233

[46] Badri H, Monsieurs P, Coninx I, Wattiez R, Leys N. Molecular investigation of the radiation resistance of edible cyanobacterium Arthrospira sp. PCC 8005. Microbiologyopen. 2015; 1–21. 10.1002/mbo3.229 25678338 PMC4398503

[47] Irizarry RA, Hobbs B, Beazer-barclay YD, Antonellis KJ, Scherf UWE, Speed TP. Exploration, normalization, and summaries of high density oligonucleotide array probe level data. biostatistics. 2003;4: 249–264. 12925520 10.1093/biostatistics/4.2.249

[48] Bolstad BM, Irizarry RA, Astrand M, Speed TP. A comparison of normalization methods for high density oligonucleotide array data based on variance and bias. Bioinformatics. 2003;19: 185–193. 12538238 10.1093/bioinformatics/19.2.185

[49] Smyth GK. Linear models and empirical bayes methods for assessing differential expression in microarray experiments. Stat Appl Genet Mol Biol. 2004;3: 25 10.2202/1544-6115.1027 16646809

[50] Benjamini Y, Hochberg Y. Controlling the false discovery rate : a practical and powerful approach to multiple testing. J R Stat Soc. 1995;57: 289–300.

[51] MacLean B, Tomazela DM, Shulman N, Chambers M, Finney GL, Frewen B, et al Skyline: an open source document editor for creating and analyzing targeted proteomics experiments. Bioinformatics. 2010;26: 966–8. 10.1093/bioinformatics/btq054 20147306 PMC2844992

[52] Schilling B, Rardin MJ, MacLean BX, Zawadzka AM, Frewen BE, Cusack MP, et al Platform-independent and label-free quantitation of proteomic data using MS1 extracted ion chromatograms in skyline: application to protein acetylation and phosphorylation. Mol Cell Proteomics. 2012;11: 202–14. 10.1074/mcp.M112.017707 22454539 PMC3418851

[53] Forchhammer K. PII signal transducers: novel functional and structural insights. Trends Microbiol. 2007;16: 65–72. 10.1016/j.tim.2007.11.004 18182294

[54] Lüddecke J, Forchhammer K. From PII signaling to metabolite sensing: A novel 2-oxoglutarate sensor that details PII—NAGK complex formation. PLoS One. 2013;8: 1–11. 10.1371/journal.pone.0083181 PMC3861474 24349456

[55] Von Wobeser EA, Ibelings BW, Bok J, Krasikov V, Huisman J, Matthijs HCP. Concerted changes in gene expression and cell physiology of the cyanobacterium Synechocystis sp. strain PCC 6803 during transitions between nitrogen and light-limited growth. Plant Physiol. 2011;155: 1445–57. 10.1104/pp.110.165837 21205618 PMC3046598

[56] Hasunuma T, Kikuyama F, Matsuda M, Aikawa S, Izumi Y, Kondo A. Dynamic metabolic profiling of cyanobacterial glycogen biosynthesis under conditions of nitrate depletion. J Exp Bot. 2013;64: 2943–54. 10.1093/jxb/ert134 23658429 PMC3697948

[57] Kolodny NH, Bauer D, Bryce K, Klucevsek K, Lane A, Medeiros L, et al Effect of nitrogen source on cyanophycin synthesis in Synechocystis sp. strain PCC 6308. J Bacteriol. 2006;188: 934–40. 10.1128/JB.188.3.934 16428397 PMC1347322

[58] Xin L, Hong-ying H, Ke G, Ying-xue S. Effects of different nitrogen and phosphorus concentrations on the growth, nutrient uptake, and lipid accumulation of a freshwater microalga Scenedesmus sp. Bioresour Technol. Elsevier Ltd; 2010;101: 5494–5500. 10.1016/j.biortech.2010.02.016 20202827

[59] Mata TM, Martins AA, Caetano NS. Microalgae for biodiesel production and other applications: A review. Renew Sustain Energy Rev. 2010;14: 217–232. 10.1016/j.rser.2009.07.020

[60] Bryant DA. The Molecular Biology of Cyanobacteria. Bryant DA, editor. Kluwer Academic Publishers; 2006.

[61] Campbell J, Stevens SE, Balkwill DL. Accumulation of poly-beta-hydroxybutyrate in Spirulina platensis. J Bacteriol. 1982;149: 361–363. 6798024 10.1128/jb.149.1.361-363.1982 PMC216630

[62] Schlebusch M, Forchhammer K. Requirement of the nitrogen starvation-induced protein s110783 for polyhydroxybutyrate accumulation in synechocystis sp. strain PCC 6803. Appl Environ Microbiol. 2010;76: 6101–6107. 10.1128/AEM.00484-10 20675451 PMC2937498

[63] Monshupanee T, Incharoensakdi a. Enhanced accumulation of glycogen, lipids and polyhydroxybutyrate under optimal nutrients and light intensities in the cyanobacterium Synechocystis sp. PCC 6803. J Appl Microbiol. 2014;116: 830–838. 10.1111/jam.12409 24299499

[64] Penning De Vries FWT, Brunsting AHM, Van Laar HH. Products, requirements and efficiency of biosynthesis : A quantitative approach. J Theor Biol. 1974;45: 339–377. 4367755 10.1016/0022-5193(74)90119-2

[65] Poorter H. Construction costs and payback time of biomass: A whole plant perspective In: Roy J, Garnier J, editors. A whole plant perspective on carbon-nitrogen interactions. Leiden: Backhuys Publishers; 1994 pp. 111–127.

